# Pullets had higher bursal and thymic weight indices and more antibody response to La Sota vaccination than broiler chickens (*Gallus gallus domesticus*)

**DOI:** 10.1002/vms3.226

**Published:** 2019-12-10

**Authors:** Amarachukwu O. Igwe, John I. Ihedioha, Didacus C. Eze, John O. A. Okoye

**Affiliations:** ^1^ Department of Veterinary Pathology Michael Okpara University of Agriculture Umudike Abia State Nigeria; ^2^ Department of Veterinary Pathology and Microbiology University of Nigeria Nsukka Enugu State Nigeria

**Keywords:** broiler, Newcastle disease, pullet, serology, vaccine

## Abstract

This study investigated the immune responses to La Sota vaccination, used in protection of chickens against Newcastle disease, in light weight type breeds of chickens (pullets) and heavy weight type breeds of chickens (broilers) used in commercial poultry production. Seven‐week‐old 50 White Marshall broilers (Br) and 50 Isa Brown pullets (Pu) were randomly divided into four groups: vaccinated broilers chickens; (VBr), unvaccinated broiler chickens (UBr), and vaccinated pullet chickens (VPu) and unvaccinated pullet chickens (UPu). Chickens in groups VBr and VPu were vaccinated with La Sota vaccine, whereas groups UBr and UPu were not vaccinated. On day 0 post vaccination (PV), six chickens from group Br and Pu, and on day 4 PV, three chickens from each four groups were sacrificed and the bursa weight index (BWI), thymus weight index (TWI) and the splenic weight index (SWI) were obtained. The chickens were observed for clinical signs and lesions. Serum samples were collected from the chickens in all the groups on days 0, 7, 14, 21, 28 PV and assayed for haemagglutination inhibition (HI) antibodies. The BWI, TWI and SWI were 0.37 ± 0.05, 0.35 ± 0.17, 0.65 ± 0.26 for pullets and 0.11 ± 0.04, 0.13 ± 0.02, 0.36 ± 0.17 for broilers on day 0 PV. On day 4 PV there was no significant difference (*p* < .05) between the indices of the vaccinated and unvaccinated chickens. The geometrical mean antibody titres (GMT) of the pullets were 2 to 3 times higher than those of the broilers on days 7 to 28 PV. Vaccination did not produce clinical signs or lesions. The above observations show that naturally pullets produce higher antibodies than broilers because of their higher BWI.

## Graphical abstract

La Sota vaccination, used in protection of chickens against Newcastle disease, did not produce clinical signs or lesions in vaccinated broiler and pullet chickens, however, pullets produce higher antibodies than broilers because of their higher bursa weight index. The above observations show that naturally pullets produce higher antibodies than broilers because of their higher BWI. More work needs to be done using other breeds of pullets and broilers to confirm this observation.

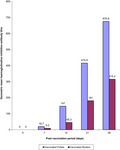

## 1 INTRODUCTION

Newcastle disease (ND) is an acute highly pathogenic viral disease of birds with worldwide distribution. It has been regarded as one of the most important diseases of birds, including domestic poultry (Alexander & Senne, 2008). This is not only due to the devastation Newcastle disease virus (NDV) infections may have on the birds infected, but also the economic impact that may ensue due to trading restrictions and embargoes placed on areas and countries where outbreaks have occurred (Alders, 2014; Aldous & Alexander, 2001). The disease is enzootic in Africa and is characterized by marked variation in morbidity, mortality, clinical signs and lesions in a variety of avian species (Cattoli, Susta, Terregino, & Brown, 2011; Igwe, Afonso, Ezema, Brown, & Okoye, 2018a; Igwe, Ezema, Eze, & Okoye, 2014; Miller & Koch, 2013). In general terms, infection of birds with any strain of NDV may be referred to as ND but because ND is a ‘notifiable disease’ to the Office International des Epizooties (OIE) due to the severe nature of the disease and the associated consequences, ND is caused only by infections with virulent strains of avian orthoavulavirus 1, commonly known as avian paramyxovirus 1, or NDV, used in this paper (Amarasinghe et al., 2019). All strains of NDV belong to the genus *Orthoavulavirus*, subfamily *Avulavirinae*, family *Paramyxoviridae* and order *Mononegavirales* (Walker et al., 2019). This enveloped virus, is pleomorphic in shape and has a single‐stranded, non‐segmented, negative sense ribonucleic acid (RNA) genome of approximately 15.2kb. The genome codes for six genes encoding for six structural proteins, from the 3′ to 5′ namely: nucleoprotein (NP), phosphoprotein (P), matrix protein (M), fusion protein (F), haemagglutinin‐neuraminidase protein (HN) and RNA‐dependent RNA polymerase (L), and an additional protein V that is expressed by RNA editing of P mRNA (Lamb & Parks, 2007).

Like all other RNA viruses, NDV is constantly evolving. Even though all strains of NDV are contained in one serotype, there are phylogenetic differences found when comparing genome relatedness. Strains are divided into two classes, class I and class II, with class II further divided into 18 genotypes (Diel et al., 2012). Class I viruses are typically isolated from wild birds and all reported strains are of low virulence except for one strain, chicken/ Ireland/1990 (Alexander et al., 1992). Class II, genotype I NDV are all of low virulence except for the virulent NDV that caused the ND outbreak in 1998 in Australia (Gould et al., 2001). NDV strains of class II, genotypes III–IX and XI–XVIII are all virulent (Courtney et al., 2012; Diel et al., 2012; Dimitrov, Ramey, Qiu, Bahl, & Afonso, 2016; Shittu et al., 2016). Alternative or complementary control strategies are required for increased host resistance to infection or disease to take care of problems that may arise due to phylogenic differences in the strains of the virus. Host genetic variation in disease resistance invariably exists, due in part to the variability in host immune responses to infection (Bishop, 2004). As humoral immunity from vaccination is critical to ND control (Kapczynski, Afonso, & Miller, 2013), another important aspect that can be a complementary control strategy is the difference in immune response due to genetic or breed variation. Due to the variability in host immune responses to infection, genetics influence both innate and adaptive immune responses and have been reported to differ between lines or genetic background of the chickens (Star, Frankena, Kemp, Nieuwland, & Parmentier, 2007) after infection or vaccination (Juul‐Madsen, Dalgaard, Rontved, Jensen, & Bumstead, 2006; Kapczynski et al., 2013; Norup et al., 2013). Genetic resistance to ND among different commercial lines within a breed (Cole & Hutt, 1961; Gordon, Beard, Hopkins, & Siegel, 1971) and among breeds of chickens have been reported (Hassan, Afify, & Aly, 2004; King, 1996), showing that breed or genetic lineage/genetics can influence the immunocompetence or immune status of the chickens. However, immune responses to La Sota vaccination among different types or breeds of chickens have not been studied. The aim of this study was to evaluate and compare the immune responses to La Sota vaccination in light weight type or breeds of chickens (pullets) and heavy weight type or breeds of chickens (broilers) used in commercial poultry production.

## 2 MATERIALS AND METHODS

### 2.1 Chickens

Fifty‐day‐old White Marshall broiler chicks and 50‐day‐old Isa Brown pullet chicks (*Gallus gallus domesticus*) procured from a reputable local commercial hatchery were used for the study. Both breeds (groups) were hatched the same day. The groupings were broiler chickens (Br) and pullet chickens (Pu). Each of the groups was brooded separately under the same environmental conditions at the departmental poultry experimental facilities. Brooding of all the chickens was done on deep litter and they were not vaccinated against any disease. Feed and water were supplied ad libitum. General care of the birds was provided in accordance with the Institutional Animal Care and Use Committee, as outlined in the *Guide for the Care and Use of Agricultural Animals in Research and Teaching* (https://www.aaalac.org/about/Ag_Guide_3rd_ed.pdf).

### 2.2 Vaccine

The La Sota vaccine used was produced by and obtained from a National Veterinary Research Institute. The vaccine was found to have a medium embryo infective dose (EID_50_) of 10^6.9^ per ml using the method of Reed and Muench (1938) and OIE (2012).

### 2.3 Experimental design

At 7 weeks of age (day 0 post vaccination), the chickens were found to be free from detectable or negative for NDV HI maternal antibody using a previously described method (OIE, 2012). They were randomly assigned into four groups of 25 chickens per breed. The groupings and their treatments were, Vaccinated Marshall broiler chickens (VBr); Unvaccinated Marshall broiler chickens (UBr); Vaccinated Isa Brown pullet chickens (VPu); Unvaccinated Isa Brown pullet chickens (UPu).

### 2.4 Vaccination of chickens

At 7 weeks of age (day 0 post vaccination), one ml of the reconstituted La Sota vaccine was administered orally by drenching each chicken in groups VBr and VPu using 5 ml syringe. Each chicken in groups UBr and UPu was drenched orally with one ml of the diluent used (unvaccinated groups) as placebo. Chickens in all the groups were observed twice daily for clinical signs for 10 days post vaccination (PV). On day 3 PV three chickens in each group were humanely sacrificed by cervical dislocation and examined for gross lesions. Samples of the bursa, spleen and thymus were fixed in 10% formal saline for 48 hr, trimmed and processed for histopathology as described (Suvarna, Layton, & Bancroft, 2018).

### 2.5 Serology

One ml of blood was collected from the jugular veins of 10 randomly selected chickens in each group, on days 0, 7, 14, 21 and 28 PV. The serum samples were harvested and assayed for haemagglutination inhibition (HI) antibodies using the method of OIE (OIE, 2012). The antigen used for the HI test was a PBS suspension of La Sota vaccine. The antibody titres were reciprocal of highest dilution of sera that gave complete inhibition of the chicken red blood cells. The Geometric Mean Titres (GMT) were calculated using the Tube Method and Table provided by Villegas (2008).

### 2.6 Bursa, thymus and spleen weight indices

The comparative effects of bursa, thymus and spleen weight indices on the antibody response to Newcastle disease (ND) La Sota vaccination in broilers and pullet chickens were also studied. On days 0 PV (before the La Sota vaccination), six chickens were randomly selected from groups Br and Pu chickens and humanely sacrificed, and on day 4 PV, three randomly selected chickens from groups VBr, UBr, VPu and UPu. The body weight, bursa, spleen and thymus weights were obtained for each chicken and the weight index of the organs was obtained for each chicken as described (Lucio & Hitchner, 1979), using the formula:

$$\text{Organ body weight index}=\frac{\text{Weight of organ}\times 100}{\text{Body weight of the chicken}}$$

### 2.7 Statistical analysis

Data generated from this study were subjected to appropriate statistical procedure using SPSS Version 15 (SPSS, 2006) as follows: On day 0 PV, the bursa, thymus and spleen weight indices of pullets were compared to those of broilers using Student's *t*‐test. On day 4 PV the indices of the vaccinated and unvaccinated pullets and broilers were subjected to one‐way analysis of variance (ANOVA) and variant means were separated using the least significant difference (LSD) method. Significance was accepted at *p* < .05. The geometric mean haemagglutination inhibition antibody titres of the vaccinated pullets and broilers were calculated and compared. Results were presented as means with standard deviations (*SD*) or geometrical mean titres as appropriate. The data were interrogated for normality.

## 3 RESULTS

### 3.1 Clinical signs and lesions

**Figure 1:**
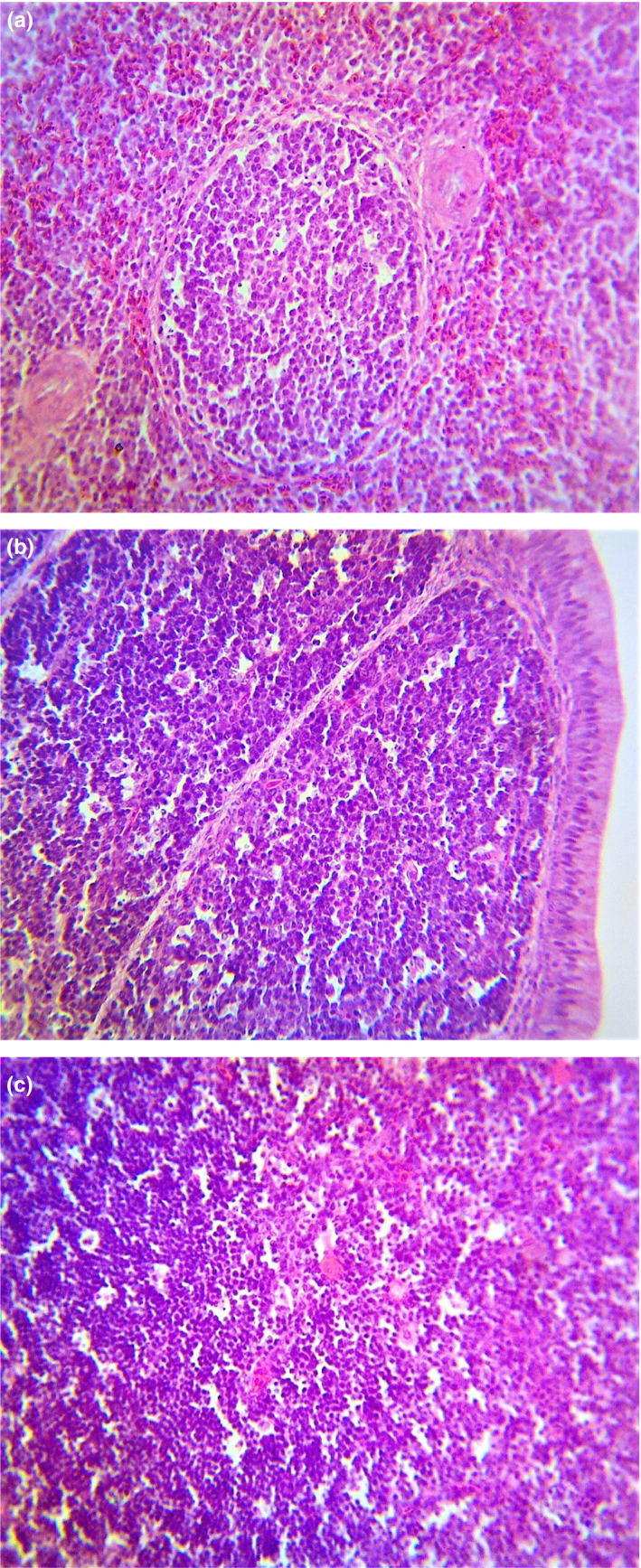
(a) Photomicrograph of spleen of vaccinated pullet showing a normal architecture on day 3 post vaccination. H & E, X400. (b) Photomicrography of bursa of Fabricius of vaccinated broiler showing a normal architecture on day 3 post vaccination, H&E, X400. (c) Photomicrography of thymus of vaccinated pullet showing a normal architecture on day 3 post vaccination, H&E, X400

No clinical signs or lesions were observed in any chicken regardless of group (Figure 1).

### 3.2 Serology

**Figure 2:**
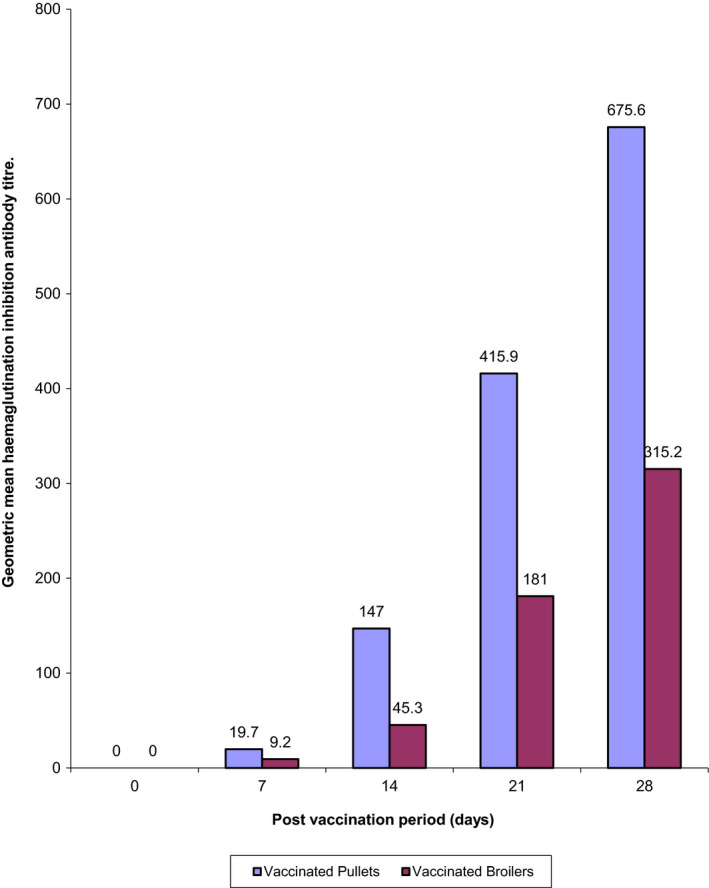
Geometric mean haemagglutination inhibition antibody titres of vaccinated and unvaccinated pullets and broiler chickens on days 0–28 post vaccination

The GMT of the HI antibody of the pullets were higher than those of the broilers on all the days assayed (Figure 2). Throughout the experiment, titres of the control groups, UPu and UBr chickens remained negative.

### 3.3 Organ bodyweight indices

The busal bodyweight index (BWI), splenic bodyweight index (SWI) and the thymic bodyweight index (TWI) were significantly (*p* < .05) higher in pullets than broilers on days 0 and 4 PV. On day 4 PV, the organ weight indices of the vaccinated chickens did not differ significantly (*p* > .05) from those of the unvaccinated (Tables 1, 2, 3, 4). The BWI and the TWI values were very close but lower than the SWI in each of the pullets and broilers assessed.

**Table 1:** Bodyweight (gm), bursa, thymus and spleen bodyweight index (%) of pullets on days 0 and 4 post vaccination

Day 0					
Mean ± standard deviation					
	S. No.	Bodyweight	Bursal Index	Spleen Index	Thymus Index
	1	600	0.31	0.81	0.32
	2	400	0.37	0.42	0.31
	3	400	0.34	0.58	0.40
	4	400	0.35	1.11	0.38
	5	400	0.44	0.50	0.30
	6	400	0.41	0.48	0.41
	Mean Index		0.37 ± 0.05	0.65 ± 0.26	0.35 ± 0.17

Day 4					
Mean ± standard deviation					
Non vaccinated pullets	S. No.	Bodyweight	Bursal Index	Spleen Index	Thymus Index
	1	400	0.36	0.74	0.47
	2	600	0.31	0.90	0.28
	3	500	0.37	0.58	0.37
	Mean Index		0.35 ± 0.05	0.74 ± 0.09	0.37 ± 0.10

Day 4					
Vaccinated pullets	S. No.	Bodyweight	Bursal Index	Spleen Index	Thymus Index
	1	400	0.22	0.35	0.21
	2	600	0.33	0.90	0.29
	3	500	0.37	0.61	0.36
	Mean Index		0.31 ± 0.09	0.62 ± 0.27	0.28 ± 0.07

**Table 2:** Bodyweight (gm), bursa, thymus and spleen bodyweight index (%) of broilers on days 0 and 4 post vaccination

Day 0					
Mean ± standard deviation					
	S/No.	Bodyweight	Bursal Index	Spleen Index	Thymus Index
	1	1,600	0.10	0.61	0.14
	2	1,400	0.19	0.24	0.14
	3	1,400	0.11	0.33	0.15
	4	1,700	0.11	0.52	0.10
	5	1,200	0.07	0.18	0.12
	6	1,400	0.11	0.30	0.14
	Mean Index		0.11 ± 0.04	0.36 ± 0.17	0.13 ± 0.02

Day 4					
Mean ± standard deviation					
Non vaccinated broilers	S/No.	Bodyweight	Bursal Index	Spleen Index	Thymus Index
	1	1,750	0.14	0.33	0.10
	2	1,700	0.10	0.59	0.19
	3	1,850	0.08	0.32	0.14
	Mean Index		0.11 ± 0.04	0.41 ± 0.15	0.14 ± 0.04

Day 4					
Vaccinated broilers	S/No.	Bodyweight	Bursal Index	Spleen Index	Thymus Index
	1	1,500	0.13	0.34	0.13
	2	1,650	0.23	0.50	0.17
	3	1,650	0.11	0.38	0.20
	Mean Index		0.16 ± 0.07	0.41 ± 0.08	0.16 ± 0.04

**Table 3:** Comparison of the bursal index, spleen index and thymus bodyweight index of pullets and broilers on day 0 post vaccination

	Mean ± standard deviation		
	Bursal Index	Spleen Index	Thymus Index
**Pullets**	0.37 ± 0.05^a^	0.65 ± 0.26^a^	0.35 ± 0.17^a^
**Broilers**	0.11 ± 0.04^b^	0.36 ± 0.17^b^	0.13 ± 0.02^b^

^ab^Different alphabetical superscript in a column indicate significant difference (*p* < .05) between the means of the pullets and that of the broilers.

**Table 4:** Comparison of the bursal index, spleen index and thymus index of vaccinated and unvaccinated pullets and broilers on day 4 post vaccination

	Mean ± standard deviation		
	Bursal Index	Spleen Index	Thymus Index
Unvaccinated Pullets	0.35 ± 0.05^a^	0.74 ± 0.09	0.37 ± 0.10^a^
Vaccinated Pullets	0.31 ± 0.09^a^	0.62 ± 0.27	0.28 ± 0.07^ac^
Unvaccinated Broilers	0.11 ± 0.04^b^	0.41 ± 0.15	0.14 ± 0.04^bc^
Vaccinated Broilers	0.16 ± 0.07^b^	0.41 ± 0.08	0.16 ± 0.04 ^c^

^abc^Different alphabetical superscript in a column indicate significant difference (*p* < .05) between the means of the vaccinated and unvaccinated pullets and that of the broilers.

## 4 DISCUSSION

The results of this experiment showed that naturally the HI antibody response to La Sota vaccination was two to three times higher in pullets than in broilers. This is likely to translate to more disease resistance in some diseases which are not acute and protective antibody response is allowed to build up before mortalities occur. Igwe, Shittu, and Okoye (2018b) reported that such resistance may not develop before mortalities occur in some acute diseases like velogenic NDV infection. King (1996) reported that the SPF White Leghorn layers were more susceptible to velogenic neurotropic NDV infection than White Rock broilers. Hassan et al. (2004) reported that the Mandarah local Egyptian breed of chicken was more resistant to virulent NDV and very virulent infectious bursal disease virus (vvIBDV) infection than other local breeds. But the antibody response and lymphocyte response to mitogen did not correlate clearly with the mortality rates showing that other genetic factors could be responsible for the disease resistance. The implication of the significantly higher bursal and thymic indices in the pullets is that the pullets will produce more humoral antibody than the broilers as already seen in this project. Cellular antibody production will also be higher in the pullets than broilers. Both organs constitute the primary or central immune system of the chicken (Sharma, 1997). The bursa produces the B lymphocytes that produce humoral antibodies or immunity, whereas the thymus produces the T lymphocytes that produce cellular antibodies or immunity (Davison, Kaspers, & Schat, 2008; Sharma, 1997). Okoye and Aba‐Adulugba (1998) and Okoye, Aba‐Adulugba, Ezeokonkwo, Udem, and Orajaka (1999) studied the BWI in light body weight chickens which included pullets, cockerel and local Nigerian chickens and the heavy types of chicken which was broilers. They observed that the light weight types had significantly higher BWI and higher mortalities at vvIBDV challenge than the heavy types and concluded that the common observations in the field where IBD outbreaks are usually very severe in pullets and cockerels but mild to moderate in broilers was due to the higher BWI and more replication of the virus in the pullets and cockerels because the bursa is the target organ in IBD. This means that those diseases where the bursa is the target organ will be more severe in the light body weight chickens than the heavy weight types. In the same way it is expected that the observations made in the pullets in this study will apply to other light weight chickens. Omeke, Ezema, Eze, and Okoye (2018) using low dose of velogenic viscerotropic NDV for challenge reported 30 and 0% mortalities in broilers and pullets respectively. They attributed the resistance of the pullets to the significantly higher NDV HI titres observed in the pullets. The result of this study therefore confirms their observation that pullets produce significantly higher NDV HI antibodies than broilers. Other factors which influence disease resistance in poultry include age, immune status of the host, genetic factors, dose and pathogenicity of the organism (Alexander & Senne, 2008). It was observed that the BWI, TWI and SWI were not affected by La Sota vaccination in this experiment. The vaccine also did not produce clinical signs or lesions. All these are expected from a lentogenic NDV infection which is mildly pathogenic. The above observations show that naturally pullets produce higher antibodies than broilers because of their higher BWI. More work needs to be done using other breeds of pullets and broilers to confirm this observation.

## 5 ETHICS STATEMENT

The authors confirm that the ethical policies of the journal, as noted on the journal's author guidelines page, have been adhered to. All animal studies were approved by the Institutional Committee on Medical and Scientific Research Ethics given by the University Committee on Medical and Scientific Research Ethics, and have therefore been performed in accordance with the ethical standards laid down in the US National Research Council's guidelines for the Care and Use of Laboratory Animals.

## CONFLICT OF INTEREST

Authors do not have any conflict of interest to report.
